# Impact and reception of point-of-care ultrasound training across medical education levels

**DOI:** 10.1186/s12909-025-06825-4

**Published:** 2025-02-17

**Authors:** Hendrik Leif Meyer, Jonas Einloft, Simon Bedenbender, Philipp Russ, Nadine Schlicker, Andre Ganser, Martin Christian Hirsch, Christian Görg, Ivica Grgic

**Affiliations:** 1 Department of Internal Medicine and Nephrology, University Hospital Giessen and Marburg, Philipps University Marburg, Marburg, Germany; 2 Institute for Artificial Intelligence in Medicine, University Hospital Giessen and Marburg, Philipps University Marburg, Marburg, Germany; 3 Interdisciplinary Center of Ultrasound Diagnostics, University Hospital Giessen and Marburg, Philipps University Marburg, Marburg, Germany

**Keywords:** Point-of-care diagnostics, Ultrasound, Clinical skills & decision support, Medical education, Artificial intelligence integration

## Abstract

**Background:**

Point-of-care ultrasound (POCUS) is a versatile and cost-effective technology that can significantly enhance medical education and clinical practice. This study examines the acceptance of POCUS among medical students and explores strategies to optimize its integration into their training.

**Materials and methods:**

A total of 318 medical students, spanning both preclinical and clinical semesters, participated in seminars that included POCUS demonstrations and hands-on practice. Their perceptions were assessed through a voluntary questionnaire based on the Technology Acceptance Model (TAM), which furthermore explored the ideal timing and potentially suitable subjects for integrating POCUS into the curriculum, with an emphasis on its role in developing clinical skills and supporting decision-making.

**Results:**

Among preclinical students, 60.7% had no prior ultrasound exposure, while 97% of clinical students reported some experience, albeit often limited. Despite the majority of senior students having some ultrasound experience, many lacked confidence in its use. Confidence in sonography skills was generally low across both groups, with 95.9% of preclinical and 79.4% of clinical students expressing a lack of confidence. Importantly, both preclinical and clinical students recognized the high usefulness of POCUS skills and rated the technology as user-friendly, with clinical students perceiving it as slightly easier to use. When asked to identify the most suitable subjects for POCUS training, students most frequently cited anatomy (35.2%) and internal medicine (16.7%). Additionally, a majority of students (57.7%) advocated for POCUS education to begin as early as the first semester.

**Conclusion:**

This study highlights a significant gap in ultrasound training among medical students, while also underscoring their strong motivation to learn and their positive perception of POCUS as a valuable tool for enhancing clinical skills and decision-making. The findings emphasize that early integration of sonography into the medical curriculum is both highly desired by students and essential for advancing medical education. This is particularly timely and important given the growing role of artificial intelligence in ultrasound technology and the opportunities expected to emerge from this integration.

**Supplementary Information:**

The online version contains supplementary material available at 10.1186/s12909-025-06825-4.

## Background

Since its inception in the mid-20th century, medical ultrasound has become an indispensable diagnostic tool across various medical specialties [1, 2]. Its numerous advantages—non-invasiveness, absence of ionizing radiation, portability, and rapid imaging capabilities—have significantly contributed to its widespread adoption [3]. As a result, basic sonography skills are now not only advantageous but increasingly expected of new medical residents in many fields [4]. In line with this trend, the German National Competency-Based Catalog of Learning Objectives in Medicine (NKLM) mandates that medical students acquire proficiency in basic sonography procedures [5].

However, integrating ultrasound education into medical curricula faces significant challenges, including the high cost of standard ultrasound devices—often exceeding US$50,000—and the constrained time available within existing medical programs [6, 7]. Point-of-care ultrasound (POCUS) devices offer a promising solution to these barriers [8]. POCUS systems, typically handheld ultrasound devices, are designed for bedside imaging and seamless integration into real-time clinical decision-making. They provide portability and accessibility for immediate diagnostic and procedural support [9]. With starting prices around US$ 3,000 for a complete system—including a probe, display screen, and software license—POCUS systems are both cost-effective and portable [10]. Furthermore, some models incorporate educational programs and AI-assisted feedback to enhance image quality and learning [11].

Efforts to integrate POCUS into medical education have gained momentum, with a growing body of research exploring diverse teaching methodologies, ranging from self-directed learning modules to tutorial-based approaches [12]. POCUS has demonstrated significant potential for use in various medical contexts, including emergency medicine, cardiac and lung ultrasound, critical care medicine, and clinical nephrology [13–18].

However, evidence regarding the effectiveness of POCUS-based ultrasound teaching remains mixed. While some studies highlight its added value in preclinical and clinical education, others do not provide clear supportive evidence [19–22]. Moreover, implementing POCUS teaching poses challenges, such as substantial purchase costs and a shortage of trained instructors, underscoring the need for targeted and efficient strategies [23]. Recent research further suggests that students may underutilize teaching programs, even when available, adding another layer of complexity to its integration [24].

Given these complexities, designing an effective curriculum requires a thorough understanding of students’ perspectives on ultrasound and POCUS devices. To this end, we engaged both preclinical and clinical students, allowing them to test POCUS devices and provide feedback on their general acceptance, potential applications, and the optimal timing for integration into the curriculum. Previous studies have shown that while students generally desire increased ultrasound training, opinions about its timing within the curriculum vary [25]. However, these studies often focus on specific student cohorts or curriculum phases, leaving broader insights underexplored [26].

## Methods

### Survey participants

Our survey included a total of 318 medical students from the Medical School of Philipps University Marburg, with 183 students in the preclinical study phase and 135 students in the clinical study phase. Participation was voluntary and conducted anonymously.

### POCUS devices and study design

In this study, we utilized the Butterfly iQ + POCUS system, a portable ultrasound probe that can be operated through connected mobile devices using the Butterfly application. These devices were more affordable than standard ultrasound machines and easier to integrate into medical education due to their compact design and portability, making them well-suited for diverse teaching environments. For this study, we used Apple iPad Mini 6th generation devices for ultrasound image visualization, connected to the probes via USB-C cable for data transfer.

To assess student acceptance of POCUS systems for sonography education, we adapted the Technology Acceptance Model (TAM), a well-established framework for analyzing user acceptance of information technologies [27–30]. The key variables of TAM include “perceived usefulness” (PU), “perceived ease of use” (PEU), and “attitude toward using” (ATU), with ATU being dependent on PU and PEU. To evaluate these variables, we developed a survey consisting of 13 closed-ended questions, each answered on a 5-point Likert scale. Additionally, we included three questions related to demographic information (age, gender, and current semester) and three questions about previous experiences with sonography. We also explored students’ perspectives on the optimal timing for initiating sonography education and the subjects into which POCUS education could be integrated. For this purpose, we included one open-ended question for subject suggestions and one closed-ended question regarding the best starting time for POCUS education, offering four predefined options and one open-ended option for alternative suggestions. A summary of the questions and their TAM assignments is provided in Supplementary Table 1.

### Implementation

First, it is important to note that the curriculum of the Medical School at Philipps University Marburg currently lacks structured, formal, hands-on sonography courses. We herein piloted POCUS education as a topic-related addition and complementary activity, respectively, embedded within existing preclinical and clinical seminars and courses. Medical students and educators first received a brief introduction to the operational aspects of the Butterfly iQ + probe and its mobile application. Following this, students were divided into groups of 5–8, each provided with a probe and an iPad. Under the supervision of a tutor, students were taught to perform an extended Focused Assessment with Sonography in Trauma (eFAST), a protocol widely used in clinical practice, especially in emergency medicine. Subsequently, students were encouraged to explore additional features of the POCUS system, including its AI features, focusing on imaging major vessels and organs, as well as testing advanced functions such as color-coded duplex ultrasound and automated bladder volume measurement (Supplementary Fig. 1). At the conclusion of the session, participants were invited to voluntarily complete printed survey forms.

### Data analysis

The survey data obtained from the printed closed-ended and open-ended questions were analyzed using the statistical software R (4.2.2) and RStudio (2023.06.1). Responses were manually digitized and entered into an Excel spreadsheet, which served as the basis for data analysis. Previous ultrasound experience was calculated from the survey data. A Likert analysis was conducted on the survey results using the likert (1.3.5) R package, with the results visualized through Likert plots generated using the ggplot2 (3.4.1) R package. To calculate summarized Likert scores for each TAM variable (PU, PEU, and ATU), the mean and standard deviation were computed for all responses related to each variable. Negatively phrased questions were inverted prior to computing the mean scores. The normality of the Likert scales (PU, PEU, and ATU) was assessed using the Shapiro-Wilk test. Subsequently, one-sample *t*-tests were conducted to determine whether the responses for each variable (PU, PEU, and ATU) were significantly different from the “neutral” response/value of 3 (i.e., “neither agree nor disagree”) [31]. To analyze and quantify responses regarding potential subjects for POCUS integration, a hybrid deductive and inductive coding approach was employed. The results were visualized using a Sankey plot, generated with the ggsankey (0.0.99999) package.

## Results

### Previous ultrasound experiences

Most preclinical students (171 out of 183) were in their 4th semester, while the majority of clinical students ( 129 out of 135) were in their 9th or 10th semesters. The German medical education system consists of a 6-year curriculum (12 semesters), with the first two years (semesters 1–4) covering preclinical subjects such as anatomy, physiology, and biochemistry. This is followed by three years (semesters 5–10) focused on clinical subjects, including internal medicine, examination courses, and surgery. The final year, known as the “practical year,” consists of three 4-month clinical rotations: one in internal medicine (mandatory), one in surgery (mandatory), and one in a specialty of the student’s choosing.

**Fig. 1:**
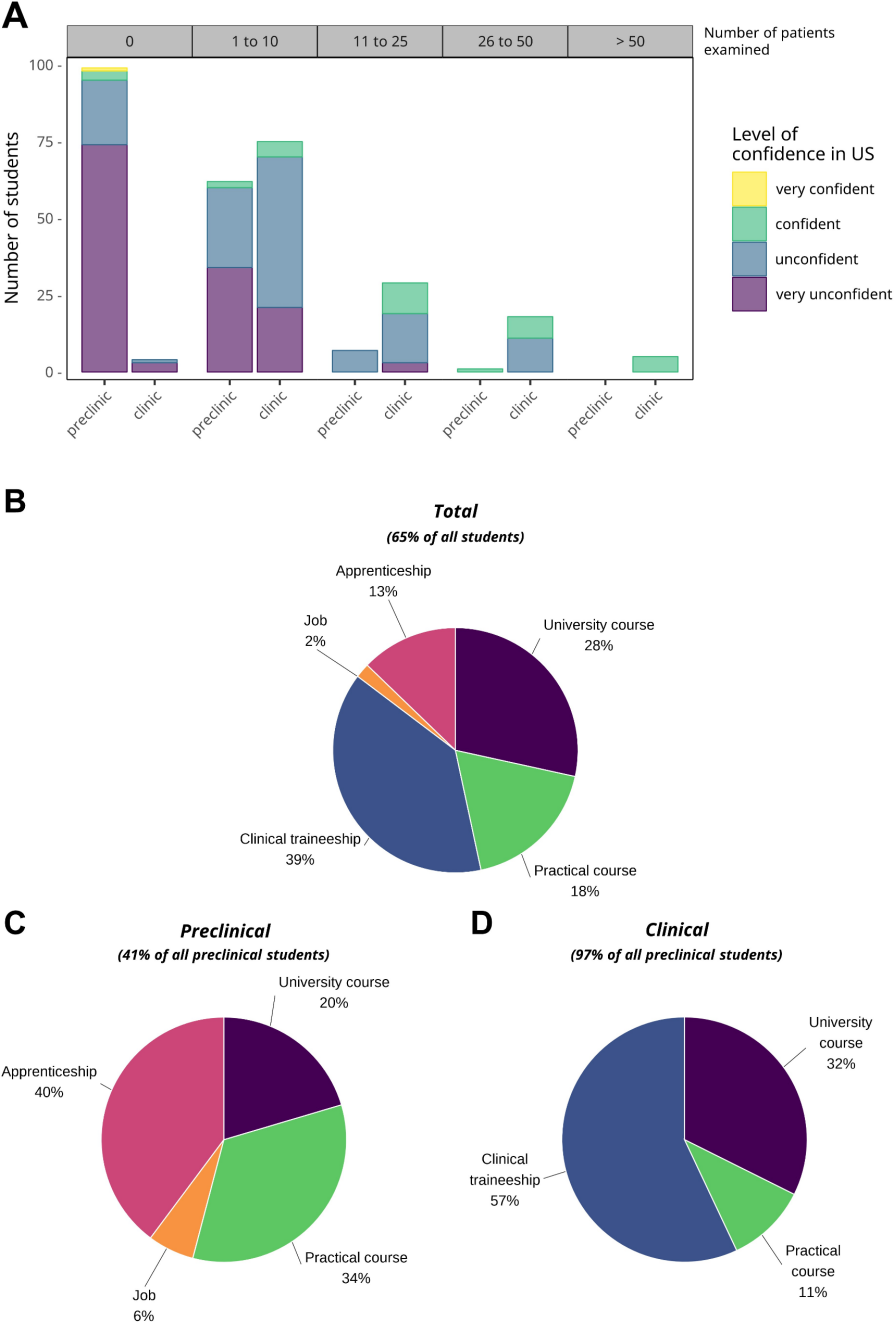
Previous Ultrasound Experiences. **a**, Students’ self-reported confidence in using ultrasound (US), correlated with the number of patients they have previously examined with ultrasound (shown horizontal axis above diagram). **b-d**, Contexts in which students have had the opportunity to use ultrasound for examinations: **(b)** overall context for all students, **(c)** context for preclinical students, and **(d)** context for clinical students

First, we aimed to assess the students’ previous experience with ultrasound, as there was no structured hands-on ultrasound training at the Medical School in Marburg. The majority of preclinical students (60.7%) reported having no prior ultrasound experience, whereas 97% of clinical students had used ultrasound to examine at least one patient (Fig. 1a, Supplementary Fig. 2a). However, 57.6% of clinical students had imaged only 1–10 patients, and 3% had no ultrasoundexperience at all. As expected, 95.9% of preclinical students felt unconfident or very unconfident in their sonography skills (Fig. 1a, Supplementary Fig. 2b). Surprisingly, 79.4% of clinical students also reported a lack of confidence, with only 20.6% feeling confident in their abilities. Even among students who had examined 1–10 patients, the majority still felt unconfident in their sonography skills.

Overall, 65% of all students had some prior ultrasound experience. To explore the context of these experiences, we asked students about the source of their exposure. Among all students, clinical traineeships accounted for approximately 40% of their ultrasound experiences, while extracurricular university courses contributed 28% (Fig. 1b). For preclinical students, apprenticeships were the most significant source of ultrasound experience (40%), followed by practical courses, which accounted for 34% (Fig. 1c). In contrast, clinical traineeships were the primary source for clinical students (57%), with extracurricular ultrasound courses contributing 32% (Fig. 1d).

### Reception and acceptance of portable POCUS systems

**Fig. 2:**
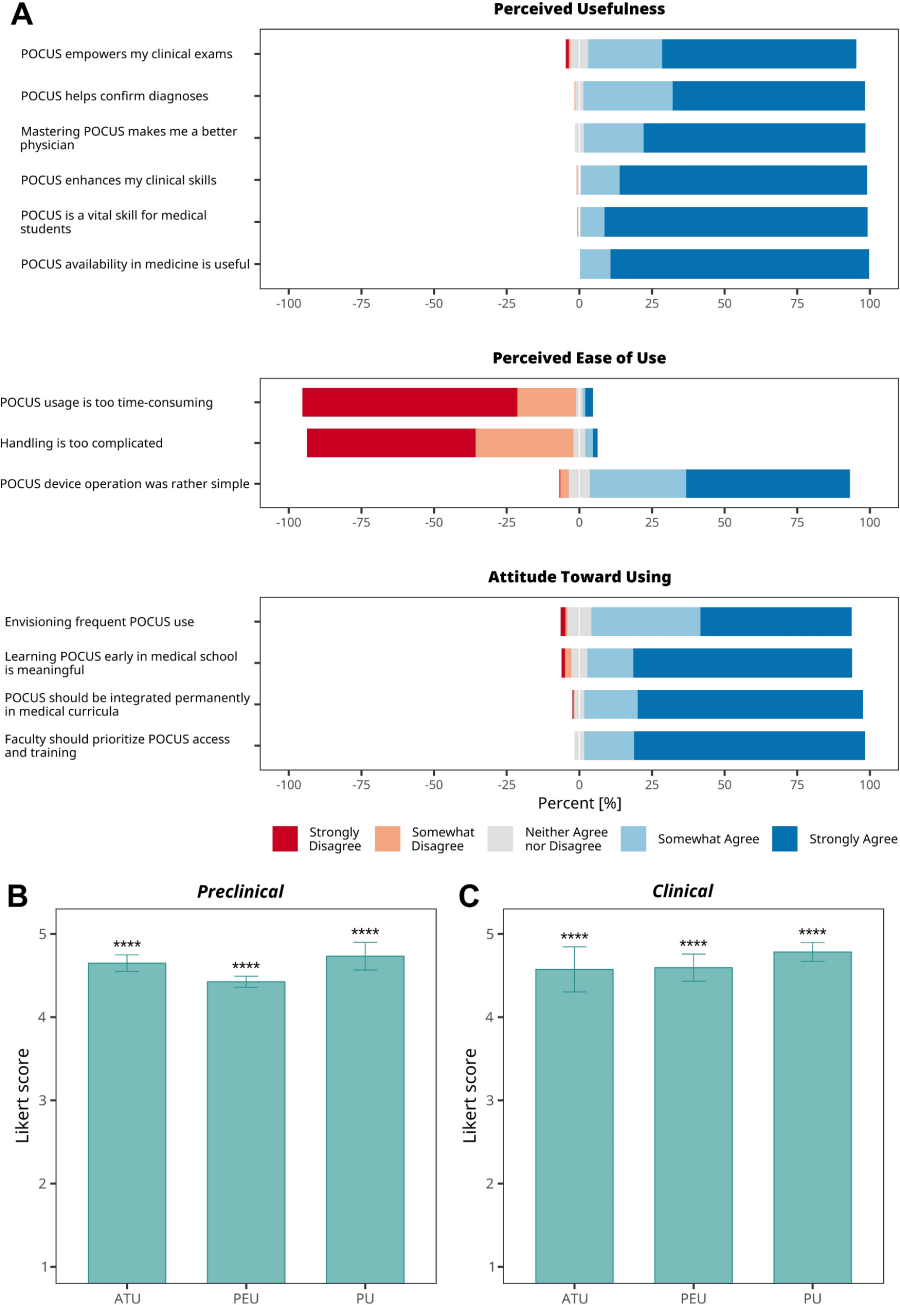
Survey Results Based on the Three Categories of the Technology Acceptance Model (TAM). **a**, Likert plots (5-point scale) displaying the survey results, with bars representing the percentage of responses for each Likert score. **b-c**, Summary of survey results for **(b)** preclinical students and **(c)** clinical students, organized by TAM categories. Mean ± standard deviation for each Likert score (5-point scale) is shown. Asterisks denote significance levels from *t*-tests comparing the means to a neutral point ($\:{\varvec{\mu\:}}_{\varvec{H0}}=3)$, indicating neither agreement nor disagreement. TAM categories include ATU (Attitude Toward Using), PEU (Perceived Ease of Use), and PU (Perceived Usefulness)

To evaluate the suitability of mobile POCUS devices for hands-on sonography courses, we applied the Technology Acceptance Model (TAM), a well-established framework used to analyze user acceptance of information technologies. The first variable we examined was “perceived usefulness” (PU), defined as the extent to which students believe that POCUS would enhance their clinical training (Fig. 2a). Both preclinical and clinical students reported highly significant and affirmative PU scores (Fig. 2b-c). A majority of students (92%) believed that POCUS would improve their clinical skills, and 97% felt that mastering POCUS would contribute to becoming better physicians. Additionally, 91% of participants strongly agreed that acquiring POCUS skills is valuable for medical students.

In addition to PU, successful implementation of a POCUS-based hands-on sonography course would likely require that students find POCUS devices easy to use. This is particularly important since more complex systems would require additional instructional time, potentially extending the duration of the sonography course. To assess this potential barrier, we examined “perceived ease of use” (PEU), defined as the degree to which students believed that using POCUS devices for sonography education would be straightforward and uncomplicated (Fig. 2a). The overall PEU score indicated a strong consensus regarding the ease of operating POCUS devices. Clinical students reported a slightly higher PEU score compared to preclinical students (4.59 ± 0.16 vs. 4.41 ± 0.06) (Fig. 2b-c). A significant majority of students (89%) found POCUS devices easy to handle, with only 3% reporting difficulties.

The third variable in the TAM is “attitude towards use” (ATU), which we defined as the degree to which students anticipated positive outcomes from using the devices. The overall ATU score demonstrated a highly significant positive attitude toward incorporating POCUS devices into hands-on sonography courses (Fig. 2). No significant differences were observed in ATU between preclinical and clinical students. Additionally, 95% of students agreed that POCUS should be a permanent part of the medical curriculum, with 78% expressing strong agreement. Despite the current structure of the preclinical curriculum, 91% of students agreed that introducing POCUS early in medical education would be beneficial. Lastly, 97% of students felt that the faculty should increase investment in the availability and instruction of POCUS.

### Students' perspectives on the implementation of POCUS education

**Fig. 3:**
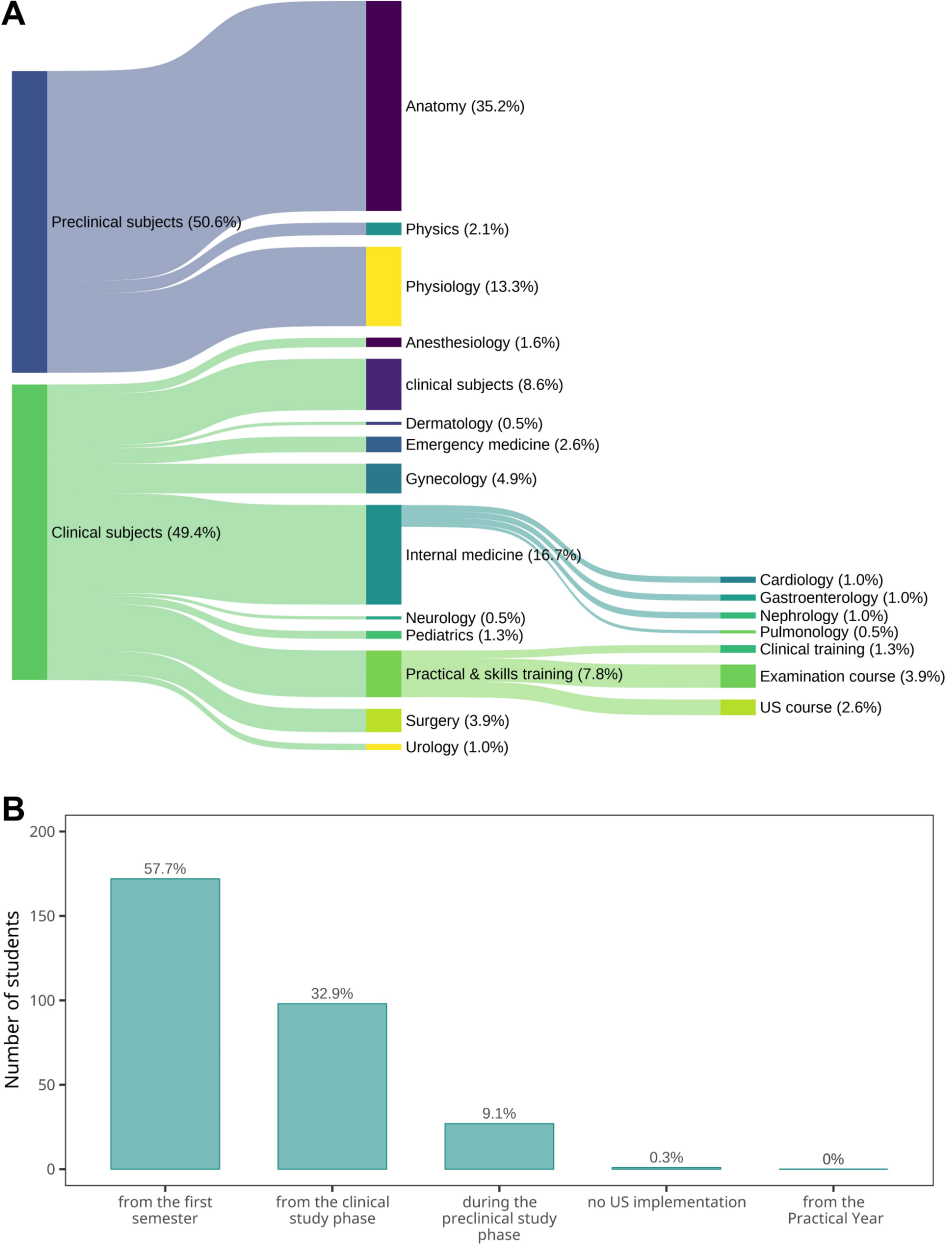
Students’ Responses Regarding the Implementation of POCUS in Medical Education. **a**, Sankey plot illustrating the distribution of students’ suggestions regarding the integration of POCUS into various medical education subjects. Responses are divided into preclinical and clinical subjects, with link widths corresponding to the frequency of mentions. Percentages denote the proportion of total responses attributed to each subject or topic. Notably, anatomy (35.2%) and internal medicine (16.7%) were the most frequently mentioned disciplines for potential POCUS application. The data underscore the perceived relevance of POCUS across a wide array of subjects. **b**, Bar graph represents the distribution of students’ recommendations for the most appropriate stage in medical education to begin POCUS training. The majority of students (57.7%) recommended introducing POCUS education as early as the first semester, while 32.9% suggested starting it during the clinical study phase. An additional 9.1% proposed beginning POCUS training at some point during the preclinical phase. Notably, there was almost no support for delaying it until the Practical Year or excluding it entirely. The data reflect a clear preference for integrating POCUS early in medical education

Finally, to effectively design a POCUS-based sonography curriculum, it is essential to consider students’ perspectives on the ideal timing for introducing POCUS education and the subjects in which its integration would have the most impact. To gather this information, we asked students two questions: which subjects they believed would benefit most from POCUS education, and when they thought POCUS should be introduced. The suggested subjects are summarized in Fig. 3a, with both preclinical and clinical subjects mentioned at similar frequencies. Overall, the most frequently suggested subjects were anatomy (35.2%) and internal medicine (16.7%).

Interestingly, students also identified physiology and physics as relevant preclinical subjects where ultrasound teaching could be beneficial. Beyond internal medicine, several clinical subjects were frequently recommended for POCUS integration, including gynecology, emergency medicine, surgery, and anesthesiology. Notably, 8.6% of students suggested that POCUS be incorporated into all clinical subjects involving sonographic examinations. Additionally, 7.8% of respondents indicated that practical training sessions, such as physical examination courses, would be suitable for POCUS integration. A small proportion (2.6%) specifically requested a dedicated ultrasound course.

Regarding the timing of POCUS education, a majority of students (57.7%) expressed a preference for introducing POCUS early, beginning in the first semester (Fig. 3b). However, 32.9% favored implementing POCUS during the clinical phase of their studies. Additionally, 9.1% of students recommended starting POCUS education in the preclinical phase, but only after acquiring sufficient foundational knowledge in anatomy.

## Discussion

Sonography has become an essential diagnostic tool across many medical fields. However, ultrasound education in Germany remains limited, despite the strong motivation among students to learn sonography. The recent advent of hand-held POCUS probes offers a promising opportunity to facilitate hands-on sonography education for medical students. These POCUS devices are significantly more affordable than conventional ultrasound machines, highly portable, and compatible with widely used mobile displays, such as tablets and smartphones.

In this study, we surveyed 318 medical students from the Medical School of Philipps University Marburg after introducing them to POCUS devices. Although most clinical students had prior experience with sonography, nearly 80% reported feeling unconfident in its use. This gap in ultrasound education has been identified in previous studies [32, 33]. Confidence in sonography is known to correlate with the time spent using ultrasound for clinical examinations [34]. Notably, students gained most of their ultrasound experience through clinical traineeships rather than extracurricular university courses, underscoring the urgent need for comprehensive hands-on ultrasound training in undergraduate medical education [21, 35].

Hand-held POCUS devices offer a viable solution to the growing need for accessible ultrasound technology in medical education [12]. In our study, students demonstrated high scores for both perceived usefulness and positive attitudes toward usingPOCUS, with no significant difference between preclinical and clinical students. The devices were also generally perceived as easy to use. Interestingly, clinical students reported slightly higher perceived ease of use, likely due to their greater prior experience with ultrasound, which enabled quicker adaptation to POCUS probes. This finding underscores the importance of introducing ultrasound education early in undergraduate medical programs, a recommendation also supported by the European Federation of Societies for Ultrasound in Medicine and Biology (EFSUMB) [36]. Moreover, the strong endorsement of POCUS integration into the curriculum from students provides a compelling argument for medical school administrators to consider. The high levels of perceived usefulness and positive attitudes revealed in our study suggest a strong demand for ultrasound education among students.

In our exploration of the potential integration of POCUS into the curriculum, we sought students’ input on the clinical subjects where POCUS could be most effectively incorporated. Students suggested a diverse range of subjects, including internal medicine, gynecology, anesthesiology, and surgery. Notably, these recommendations align with the competencies outlined in the German National Competence-based Learning Objectives Catalogue for Medicine (NKLM), which advocates for ultrasound education in disciplines such as anesthesiology, surgery, ENT, internal medicine, pediatrics, and urology [5]. Furthermore, many students emphasized the importance of incorporating POCUS across all relevant clinical disciplines and expressed a strong desire for a dedicated practical ultrasound course, highlighting the critical need for hands-on ultrasound training in undergraduate medical education.

We also explored students’ opinions on the optimal timing for initiating ultrasound education. Notably, the majority favored starting in the first semester, despite the heavy workload and stress typically associated with the early stages of medical study [37, 38]. In contrast, about one-third of students preferred to begin ultrasound education during the clinical phase of their studies. Both preclinical and clinical students advocated for integrating ultrasound education into anatomy and physiology courses, recognizing the educational benefits of early exposure. This finding aligns with recent research supporting early and enhanced anatomical education, including the use of advanced technological tools, as a means to bridge knowledge gaps effectively [39]. The advantages of introducing ultrasound in the preclinical phase are well-documented [40–42], particularly in enhancing anatomy instruction. Ultrasound imaging allows students to simultaneously identify anatomical structures in illustrations, cadavers, and real-time ultrasound images, thereby reinforcing and deepening their understanding.

Our study has several critical limitations. While it focuses on students’ perspectives regarding POCUS-based ultrasound teaching, it does not address essential questions, such as whether this teaching method results in measurable knowledge gains—questions that require further investigation through controlled studies. Furthermore, we were unable to identify which specific teaching approaches are most effective for this purpose. Additionally, the potential influence of confounding factors cannot be overlooked. These include prior experience with ultrasound devices (e.g., during clinical rotations), individual learning styles, familiarity with technology, and group dynamics.

Despite these limitations, the large number of participants, along with the inclusion of both preclinical and clinical students, provided valuable insights into student experiences and perspectives. These findings offer important guidance for the targeted and effective integration of POCUS into medical curricula. Furthermore, our approach and reported experiences may resonate with stakeholders at other medical schools worldwide that have yet to prioritize ultrasound education. By serving as a practical template, this study could facilitate the stepwise implementation of such programs, fostering broader adoption of POCUS in medical training.

## Conclusion

In conclusion, our study underscores students’ positive perception of practice-oriented learning, highlighting the suitability of relatively low-cost hand-held POCUS devices for this approach. Students strongly advocate for introducing POCUS-based teaching early in the preclinical semesters and have identified diverse topics for its application across both preclinical and clinical phases. These insights serve as a call to action, offering valuable guidance for future curriculum development. To advance the integration of POCUS into medical education, further research is essential to identify optimal implementation strategies. Key priorities include comparing POCUS-supported teaching with conventional methods to assess its effectiveness, determining whether the course should be optional or mandatory, and evaluating the respective roles of student-led tutoring and faculty supervision. Additionally, the potential of emerging AI-driven tools to enhance the learning experience and improve image data interpretation warrants exploration. Addressing these areas will help refine POCUS integration and maximize its educational impact.

## Electronic supplementary material

Below is the link to the electronic supplementary material.

Supplementary Material 1

## Data Availability

Data is provided within the manuscript or supplementary information files.
