# Supplementary material for: Impact and reception of point-of-care ultrasound training across medical education levels
Source: BMC Med Educ. 2025 Feb 17;25:255. doi: 10.1186/s12909-025-06825-4 (PMC11834199; doi:10.1186/s12909-025-06825-4)
Supplement: Supplementary file 1 — Supplementary Material 1 [file 12909_2025_6825_MOESM1_ESM.docx]

Supplementary Material

Impact and Reception of POCUS training across German Medical Education Levels

Supplementary Table 1 | Survey design.

| **Category** | **Question type** | **Question text** |
| --- | --- | --- |
| Perceived Usefulness (PU) | Likert (5 point) | I believe that learning POCUS would be a valuable skill for medical students.  I feel encouraged to use POCUS in clinical examination.  I think POCUS can help improve my clinical skills.  I think POCUS can be helpful to confirm or rule out possible differential diagnoses.  Confident mastery of POCUS is a skill that will help me be a better physician.  I think the availability of POCUS in medicine is useful. |
| Perceived Ease of Use (PEU) | Likert (5 point) | It was comparatively easy for me to understand the handling/use of the POCUS devices.  I think the handling of POCUS is too complicated. *  POCUS will not be of interest for me, as its usage is too time-consuming for me. * |
| Attitude Toward Using (ATU) | Likert (5 point) | I can see myself using POCUS regularly in my future clinical practice.  In my opinion, POCUS should be a permanent part of the medical school curriculum.  I think it makes sense to learn about POCUS early in medical school (e.g., anatomy semester).  I strongly believe that the faculty should invest more in making POCUS more available and competent in medical school. |
| Potential Application of VR | Open-ended  Multiple Choice/Open Ended | I would like to see POCUS used in the following courses:  When should POCUS be used in teaching? |

* Negatively phrased questions were inverted prior to computing the mean score.


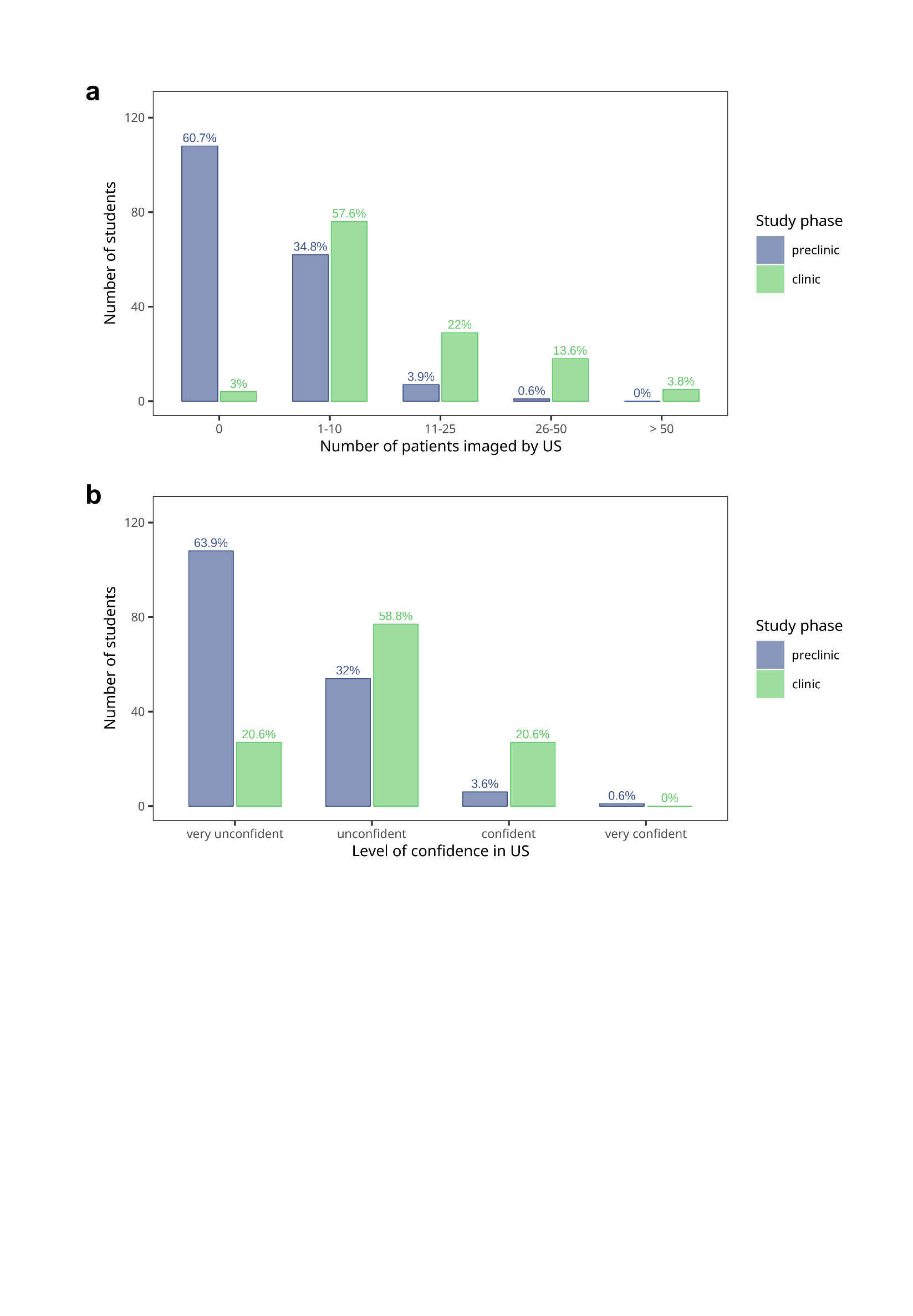


Supplementary Figure 1 | Previous US Experience and Level of Confidence. a, Percentage of students for each level of previous US experience measured by the number of patients already imaged by US. b, Percentage of students for each self-reported level of confidence.

Supplementary Figure 2 | Implementation of Hands-on POCUS courses.
